# Fine Grinding or Expanding as Pre-treatment for Pelleting in Processing Diets Varying in Dietary Rapeseed Expeller Proportions: Investigations on Performance, Visceral Organs, and Immunological Traits of Broilers

**DOI:** 10.3389/fvets.2020.550092

**Published:** 2020-10-14

**Authors:** Wendy Liermann, Jana Frahm, Andreas Berk, Verena Böschen, Sven Dänicke

**Affiliations:** ^1^Institute of Nutritional Physiology “Oskar Kellner“, Leibniz Institute for Farm Animal Biology, Dummerstorf, Germany; ^2^Institute of Animal Nutrition, Friedrich Loeffler Institute, Federal Research Institute for Animal Health, Brunswick, Germany; ^3^Research Institute of Feed Technology of the International Research Association of Feed Technology e.V., Brunswick, Germany

**Keywords:** broiler performance, gastrointestinal tract, feed technology, ascites, T and B cells, rapeseed

## Abstract

Pelleted feed is associated with improved broiler performance but also with a higher incidence of proventricular dilatation and ascites. The present study aimed to investigate influences of expanded and pelleted (ExP) or finely ground and pelleted feeds (FgP) containing either 6% rapeseed expeller (RSE) or 12% RSE on these adverse effects by studying performance, visceral organ, and immunological traits in 36 broilers. ExP reduced daily feed intake compared to FgP when feeding a 6% RSE diet (*P* < 0.05) but did not affect the daily feed intake when feeding a 12% RSE diet, which was also reflected in the body weight gain. There were no significant differences in the size of proventriculus and gizzard between feeding groups but significant diet-by-technical feed treatment interactions in case of proventricular and gizzard weights and the proventricular length (*P* < 0.05). Proventriculi and gizzards were heavier in birds fed 6%ExP than proventriculi or gizzards of animals from all other groups except for birds of the group 12%FgP. A total of three animals (1 from 6%ExP, 1 from 6%FgP, and 1 from 12%ExP) developed ascites during the study. Pooled LsMeans of peripheral blood leucocyte proportions of CD3^+^/CD4^−^/CD8^−^ cells were increased in birds fed FgP compared to birds fed ExP (*P* = 0.048). Pooled LsMeans of CD3^+^/CD4^+^/CD8^+^ T cell subsets in jejunal *lamina propria* were higher in birds fed 12% RSE compared to birds fed 6% RSE (*P* = 0.024). Concluding, technical feed treatment or diet did not inhibit adverse effects of pelleting on gizzard and proventricular development. Morphometric alterations of proventriculus and gizzard might modify the local immune system of the distal digestive tract and promote the development of ascites; however, further studies are required to confirm this hypothesis since in the present study only three birds developed ascites.

## Introduction

Pelleting is an important processing method that merges small particles to larger particles (1). Thus, this technique plays a key role for storage suitability and transportability of feeds, the inhibition of feed component separation, and avoidance of feed selection by the animals (2, 3). Further studies also give evidence that the compaction process has stimulating effects on daily feed intake and body weight (BW) gain in broilers (4, 5). In addition to the positive effects on broiler performance, negative effects of pellet feeding were reported in broiler studies such as proventricular dilatations and an inhibited development of the gizzard (4–6). In studies of Liermann et al. (5) it was indicated that these alterations also impact the down-stream digestive tract and the local immune system of broilers. Furthermore, feeding of pelleted feed is associated with a higher incidence of ascites (7, 8). Various pre-treatments of feed can contribute to a successful pelleting process (3, 9). Two common technical pre-treatments are fine grinding and expander treatment. Inter alia, they influence the pellet hardness and durability, which are important for pellet quality and texture (9, 10). Parsons et al. (11) showed that both the quality and texture of pellets influence broiler performance. Additionally, the pre-treatments allow processing of even difficult to pellet feed stuffs (2, 9). Moreover, hydrothermal processing methods such as expander treatment are hygienization-methods and able to degrade some anti-nutritive substances (10).

The current experiment studied whether the mentioned pre-treatments can possibly exacerbate or cancel nutritional disadvantages of pellet feeding in broiler fattening. Two different diet types were used that differed in their rapeseed expeller (RSE) content to study the possible interactions between technical feed treatment (TFT) and diet composition. RSE is an important protein source but also known to contain anti-nutritive substances such as glucosinolates (12). The current study also investigates the impacts of the aforementioned diet alterations on the proximal digestive tract and immunological traits in more detail to understand the impact of these modifications on the health of broilers.

## Materials and Methods

The current study was conducted in the experimental station of the Institute of Animal Nutrition, Friedrich-Loeffler-Institut, Federal Research Institute for Animal Health in Brunswick, Germany. The housing conditions and sample collection during the experimental period were in accordance with the German animal protection law guidelines and were approved by the Lower Saxony State Office for Consumer Protection and Food Safety, LAVES, Germany (registration number: 33.9-42502-04-082/09).

### Experimental Design and Animals

Thirty-six male broilers (Ross 308) were allocated to four feeding groups (nine birds per group) immediately after hatching. Feeds differed both in composition and in processing as shown in Table 1. Briefly, two different diet types were formulated according to the requirements of the GfE (13), which contained either 6% RSE or 12% RSE (Table 2). After mixing of the complete feeds they were either finely ground or expanded. For fine grinding a hammer mill (Tietjen Verfahrenstechnik GmbH, Hemdingen, Germany) with a 3 mm screen size was used. Expander treatment was realized by an expander from the Amandus Kahl GmbH & Co.KG (Reinbek, Germany) at 130°C and 3% steam supply. Depending on throughput, the retention time in the expander was 5–8 s. Subsequently, all feeds were pelleted (pellet-die press, Salmatec, Gödenstorf, Germany). Finely ground feeds were pelleted at 75°C and expanded feeds at 87°C and 3% steam. The retention time in the pellet press depended on throughput and varied between 4 and 10 s. The steam supply during hydrothermal procedures was controlled by a DVHP flow measuring system (Heinrichs Messtechnik GmbH, Köln, Germany). Manufactured pellet products had a diameter of 3 mm.

**Table 1:** Experimental design.

**Feeding group**	**Percentage of rapeseed expeller [%]**	**Technical feed treatment**
6%ExP (*n* = 9)	6	Expanded and pelleted
6%FgP (*n* = 9)	6	Finely ground and pelleted
12%ExP (*n* = 9)	12	Expanded and pelleted
12%FgP (*n* = 9)	12	Finely ground and pelleted

**Table 2:** Feed composition, feed particle size, and pellet abrasion.

**Variable**	**6% rapeseed expeller**		**12% rapeseed expeller**	
	**ExP^1^**	**FgP^2^**	**ExP**	**FgP**
Ingredients, g/kg feed				
Corn	240		240	
Wheat	364		339	
Wheat middlings	32		32	
Hi-pro soybean meal	237		202	
Soybean oil	14		11	
Rapeseed expeller	60		120	
Rapeseed oil	22		26	
Calcium carbonate	12		12	
Calcium-sodium-phosphate	4		3	
Mono-calcium-phosphate	3		3	
Premix^3^	12		12	
Calculated composition				
Metabolizable energy, MJ/kg feed	13		13	
Crude protein, g/kg feed	205		205	
Lysine, g/kg feed	11.8		11.8	
Methionine, g/kg feed	5.5		5.5	
Calcium, g/kg feed	8.5		8.5	
Phosphorus, g/kg feed	5.5		5.5	
Feed particle size before hydrothermal treatment				
> 710 μm, %	81.5	53.6	74.5	46.8
710–125 μm, %	17.7	45.9	24.5	52.9
<125 μm, %	0.8	0.5	1.1	0.3
D50^4^	1.9	0.8	1.5	0.7
Pellet abrasion, %	6.0	3.9	4.4	4.4

1 * Expanded and pelleted*.

2 * Finely ground and pelleted*.

3 * Delivers per kg feed: 12,000 IU vitamin A; 119 μg cholecalciferol; 60 mg Vitamin E; 60 mg iron; 11.3 mg copper; 67.5 mg zinc; 75 mg manganese; 1.5 mg iodine; 0.23 mg selenium; 1,220 U xylanase; 152 U glucanase; 750 U phytase*.

4 * Cumulative particle size distribution at 50%*.

### Feeding Management and Husbandry

Until the 14th day after hatching birds were housed in floor pens and in groups on chopped straw. Thereafter, they were housed in individual balance cages without bedding to enable the determination of the individual daily feed intake. The floor pens and balance cages were integrated in climate controlled rooms. The illumination period lasted from 0000 h until 2300 h on the first 2 days after hatching and from day three on from 0400 h until 2000 h. Every second day the ambient temperature was decreased by 1°C from 36°C on day 1 after hatching to 22°C on day 28 after hatching. The different feeds were available *ad libitum* in dry form from the first day of life until slaughter. However, on the first 14 days after hatching the pellets were fed crumbled. Floor pens were equipped with bell drinkers (only the first 5 days after hatching) as well as nipple drinkers and balance cages with a water trough. Thus, birds had free access to water over the entire experimental period. On day 8 after hatching broilers were vaccinated against Newcastle disease virus.

### Data and Sample Collection

From the 14th day after hatching broilers were weighed and the individual feed intake was recorded weekly. Furthermore, the daily water consumption was determined representatively from day 21 until day 25 after hatching. Therefore, the water troughs were filled with 800 ml of water in the morning. The reduction of water was measured 24 h after trough filling. To determine the evaporation loss a reference trough was positioned in the middle of the room.

On day 28/29 (18 birds per day) after hatching broilers were weighed and body temperatures were measured. Blood samples were collected for the determination of blood gases from the *Vena jugularis dexter* in a syringe containing 10 μl Heparin-Natrium Braun 25.000 I.E. solution (B. Braun Melsungen AG, Melsungen, Germany). Thereafter, birds were slaughtered by mechanical stunning and exsanguination. During exsanguination additional blood samples were collected from neck vessels in conventional EDTA and serum tubes. Heart, liver without bladder, spleen, bursa of *Fabricius* and pancreas were dissected and weighed. Furthermore, the proventriculus and the gizzard were separated, emptied, and weighed individually. Additionally, the length and width of both organs was measured by a caliper as well as the thickness of main muscles of gizzard as described by Liermann et al. (5). The jejunum was separated between the duodenum and the Meckel's diverticulum. The tissue was rinsed gently with PBS and stored in CUSTODIOL® (Dr. FRANZ KÖHLER Chemie GmbH, Bensheim, Germany) on ice. Moreover, caecal tonsils were dissected and stored in PBS on ice until further analyses.

### Feed Analyses

Particle size distribution of the complete initial feeds and finely ground feeds was determined by dry sieve analyses according to DIN 66165-1:1987-04 and DIN 66165-2:1987-04 (14, 15) and by using a sieve tower corresponding to DIN ISO 3310/1 (16) before hydrothermal treatment.

The pellet abrasion was estimated by the standard method of the American Society of Agriculture Engineers (17).

### Hematology, Oximetry, and Clinical Chemistry

For the determination of the hematocrit heparinized blood was collected in a micro-hematocrit tube and centrifuged at 12,000 × g by a micro-hematocrit centrifuge (Haematokrit 210; Hettich Lab Technology, Tuttlingen, Germany).

According to Pendl (18) blood smears of EDTA blood were prepared (two per bird), stained with Pappenheim solution, and counted. Leucocytes and thrombocytes were counted in 20 fields of vision (at 1,000 × magnification) and at least 200 leucocytes were differentiated according to their morphological characteristics.

Blood gas traits (pCO_2_; pO_2_; TCO_2_; BE_ecf_; ${\text{HCO}}_{3}^{-}$), blood pH, blood electrolytes (sodium, potassium, chloride, ionized calcium), and the metabolites glucose and lactate were determined by the analyzer GEM® Premier 4,000 (Instrumentation Laboratory, Munich, Germany) in heparinized blood. Further metabolites and liver enzymes were analyzed by an automatic clinical chemistry analyzer in blood serum (Eurolyser CCA180, Eurolab, Austria).

### Isolation of Leucocytes From Different Localizations

The isolation methods of peripheral blood lymphocytes (PBL) and leucocytes from *lamina propria* and caecal tonsils were described in a previous study of Liermann et al. (5) and are briefly summarized in the following subsections.

### Isolation of PBL

A total of 1 ml EDTA blood was diluted with PBS (1 ml), covered with 3 ml Biocoll separation solution (Biochrom AG, Berlin, Germany), and centrifuged at 681 × g for 12 min, without break, at room temperature. Cells were isolated from the interphase, resuspended in PBS, and centrifuged once again at 235 × g for 10 min without break at 4°C. The supernatant was discarded. The received pellet was resuspended in 500–1,000 μl HEPES buffered saline and stored on ice until further analyses.

### Isolation of Leucocytes From Caecal Tonsils

Caecal tonsils were rinsed and surrounding fat tissues were cut. The rinsed caecal tonsils were transferred into a petri dish containing 5 ml PBS and cut lengthwise. Cells were released by gentle scraping using a scalpel. The received cell suspension was sieved using CellTrics® (mash size 50 μm) (Partec GmbH, Görlitz, Germany) and washed by HEPES buffered saline and centrifuged at 250 × g for 5 min at 4°C. The pellet was resuspended in 500–1,000 μl HEPES buffered saline and stored on ice until further analyses steps.

### Isolation of Leucocytes From Jejunal *Lamina propria*

Firstly, the surrounding connective tissue was removed as completely as possible from the jejunum. Thereafter, the jejunum was stored on ice for 20 min in a beaker containing PBS. After the resting period the jejunum was opened and cut into small pieces, which were transferred into Erlenmeyer flasks. A total of 50 ml HEPES buffered, Hanks balanced salt solution (without Ca^2+^ and Mg^2+^; Biochrom GmbH, Berlin, Germany) and a magnetic stir bare were added to the flasks. The samples were stirred at room temperature and after 10 min the HEPES buffered, Hanks balanced salt solution was renewed. The washing steps were repeated twice. In the meantime, 25 ml Roswell Park Memorial Institute medium (RPMI-1640; Biochrom GmbH, Berlin, Germany) was heated to 37°C. A total of 180 U collagenase type V (Sigma-Aldrich, Chemie GmbH, Munich, Germany) was added to the RPMI-1640. The resulting solution was transferred into the Erlenmeyer flasks containing the jejunal tissues after the last washing step. After 10 min incubation time the fluid fraction of the samples was collected in 50 ml tubes and the RPMI-1640-collagenase-mix was renewed. This step was repeated twice. Thereafter, samples were incubated with heated RPMI-1640 containing 260 U collagenase. After 15 min the resulting supernatant was also collected. Each collected cell suspension was stored on ice during the remaining incubation periods. Thereafter, both 50 ml tubes containing the cell suspensions were centrifuged at 191 × g for 10 min at room temperature. The pellet was resuspended in 25 ml RPMI-1640. The samples of the individual incubation steps were combined and centrifuged once again. A total of 25 ml of a 30% Percoll gradient (Sigma-Aldrich Chemie GmbH, Munich, Germany) was added to the resulting pellet. The samples were mixed carefully and centrifuged at 350 × g for 15 min at room temperature. The pellet was resuspended in 5 ml RPMI-1640 and the solution was sieved by Cell Trics®. Until further analyses the cell suspension was stored on ice.

### T and B Cell Phenotyping

For T and B cell phenotyping, cells isolated from blood, *lamina propria*, and caecal tonsils were stained with antibodies for CD3 (T cells), CD4 (T helper cells), and CD8 (T cytotoxic cells) (mouse anti-chicken CD3: PE; mouse anti-chicken CD4: FITC; mouse anti-chicken CD8: Cy5) according to methods described by Liermann et al. (5). Furthermore, additional samples of PBL's and cells isolated from *lamina propria* were incubated with antibodies for Bu1 (B cells) (mouse anti-chicken Bu-1: FITC; Southern Biotech; Birmingham, USA). For corresponding isotype controls either mouse IgG1 negative control: PE; mouse IgG1 negative control: FITC, or mouse IgG1 negative control: Cy5 (Southern Biotech; Birmingham, USA) were used. T and B cell subsets were measured by FACS Canto II (BD Bioscience, San Jose, USA). Therefore, at least 10,000 cells were considered. The evaluation of results and the compensation of non-specific signals indicated by the isotype controls were conducted by using the BD FACSDiva™ Software (BD Biosciences, San Jose, USA).

### Calculations and Statistical Analyses

Parameters of broiler performance and water consumption were calculated as follows:

Daily feed intake [g] = feed consumption [g]/duration of feeding period [d]

BW gain [g/d] = (final BW [g]–initial BW [g])/duration of feeding period [d]

Feed to gain ratio (FGR) [g/g] = daily feed intake [g]/BW gain [g]

Water consumption [mL/kg BW] = ((initial water volume of trough [mL]–final water volume of trough after 24 h [mL])–(initial water volume of reference-trough [mL]–final water volume of reference-trough after 24 h [mL]))/BW [kg]

Water consumption [mL/g feed intake] = ((initial water volume of trough [mL]–final water volume of trough after 24 h [mL])–(initial water volume of reference-trough [mL]–final water volume of reference-trough after 24 h [mL]))/daily feed intake [g]

Parameters of performance and water consumption of the overserved period were averaged for the observed period.

According to Pendl (18) total counts of leucocytes and thrombocytes were calculated by summation of cells counted in 20 fields of vision of a blood smear and multiplying by the factor 875. Because all measured hematocrit values were lower than 35%, leucocyte and thrombocyte counts were corrected according to the corresponding hematocrit of the bird (18).

The H/L ratio was defined as the quotient between counted heterophilic granulocytes and lymphocytes of a blood smear.

The anion gap was calculated according to the equation of Haßdenteufel and Schneider (19): Anion gap = (Na^+^ + K^+^)–(Cl^−^ + ${\text{HCO}}_{3}^{-}$).

Statistical analyses were conducted by using the MIXED procedure of SAS Enterprise Guide 6.1. The created model included the fixed effects “diet” (RSE content) and “TFT” as well as their interactions. Least squares means (LsMeans) and standard errors were estimated for each fixed factor. The differences between LsMeans were subsequently verified by the Tukey–Kramer test. At *P* < 0.05 differences were assessed as significant. Correlation coefficients according to Pearson were estimated using the SAS Enterprise Guide 6.1 and assessed as significant at a *P* < 0.05. Principal component analyses were conducted by JMP 13.1.0 to visualize relationships between variables and cases in a two-dimensional space based on correlations. The fixed factors were included as additional variables.

## Results

There were no premature animal losses during the experimental trial. The body temperature ranged between 41.0°C and 41.8°C and did not differ between feeding groups (*P* > 0.05) (data not shown). During slaughtering it was observed that three animals developed ascites. These animals belonged to the groups 6%ExP, 6%FgP, and 12%ExP.

### Performance and Water Consumption of Broilers

TFT significantly affected daily feed intake and BW gain (*P* < 0.01) (Table 3). Moreover, a significant diet-by-TFT interaction was detected in case of daily feed intake, BW gain, and FGR (*P* < 0.05). The daily feed intake and the BW gain were significantly lower in the 6%ExP group compared to the 6%FgP group (*P* < 0.01). Additionally, the BW gain was significantly reduced by feeding finely ground and pelleted feed containing 12% RSE compared to finely ground and pelleted feed containing 6% RSE (*P* = 0.025). There were no significant differences between the FGR of different feeding groups but 6%ExP and 12%FgP tended to higher FGR compared to 6%FgP and 12%ExP (*P* < 0.1).

**Table 3:** Performance and water consumption (LsMeans, *n* = 9).

**Variable**	**6% rapeseed expeller**		**12% rapeseed expeller**		**SE**	***P*****-value**		
	**ExP^1^**	**FgP^2^**	**ExP**	**FgP**		**Diet**	**TFT^3^**	**Diet x TFT**
ADFI^4^, g	75^b^	99^a^	84^ab^	86^ab^	4.1	0.650	0.003	0.010
BWG^5^, g/d	49^b^	70^a^	60^ab^	57^b^	3.1	0.672	0.006	0.001
FGR^6^, g/g	1.53	1.42	1.43	1.53	0.03	0.974	0.941	0.001
Water consumption, mL/kg BW^7^/d	205^b^	241^ab^	300^a^	251^ab^	24	0.037	0.782	0.094
Water consumption, mL/g feed intake/d	1.55^b^	1.86^ab^	2.35^a^	1.87^ab^	0.18	0.033	0.629	0.035

1 *Expanded and pelleted*.

2 *Finely ground and pelleted*.

3 *Technical feed treatment*.

4 *ADFI, average daily feed intake*.

5 *Body weight gain*.

6 *Feed to gain ratio*.

7 *Body weight*.

a, b *Different superscripts within a row mark significant differences between feeding groups (P <0.05)*.

The water consumption per kg BW and per g feed intake was mainly affected by the diet composition (*P* < 0.05). However, there was also a significant interaction between both fixed effects considering the water consumption per g feed (*P* = 0.035). The water consumption related to BW or feed intake was significantly increased in animals fed expanded and pelleted feed containing 12% RSE compared to animals fed expanded and pelleted feed containing a lower amount of RSE (*P* < 0.05).

### BW on Slaughtering Day, Visceral Organ Weights, and Morphometric Characteristics of Digestive Organs

On slaughtering day the BW of broilers fed finely ground and pelleted feed containing 6% RSE was significantly increased compared to broilers fed the other feeds (*P* < 0.05) (Table 4). Furthermore, the BW of birds fed 12%ExP was significantly higher compared to birds fed 6%ExP on slaughtering day (*P* = 0.043).

**Table 4:** Bird final body weight, organ weights and morphometric characteristics of proventriculus and gizzard (LsMeans, *n* = 9).

**Variable**	**6% rapeseed expeller**		**12% rapeseed expeller**		**SE**	***P*****-value**		
	**ExP^1^**	**FgP^2^**	**ExP**	**FgP**		**Diet**	**TFT^3^**	**Diet x TFT**
Body weight (BW), g	994^c^	1,364^a^	1,176^b^	1,123^bc^	4.6	0.531	0.002	<0.001
OW^4^, g/kg BW								
Heart	6.69^a^	6.05^ab^	6.49^ab^	5.80^b^	0.20	0.266	0.002	0.907
Liver	22.7^b^	23.4^ab^	27.8^a^	23.2^b^	1.2	0.049	0.112	0.035
Spleen	0.93	0.77	0.73	0.91	0.06	0.651	0.878	0.012
Bursa of *Fabricius*	3.08^a^	2.43^ab^	2.26^b^	2.57^ab^	0.20	0.100	0.411	0.022
Pancreas	2.71	2.48	2.45	3.00	0.15	0.402	0.280	0.015
Gizzard	15.1^a^	10.7^c^	12.5^bc^	13.4^ab^	0.6	0.903	0.008	<0.001
Proventriculus	4.89^a^	3.95^b^	3.92^b^	4.70^a^	0.16	0.508	0.643	<0.001
Gizzard/proventriculus, g/g	3.09	2.71	3.20	2.90	0.14	0.267	0.018	0.772
Section extent								
Proventricular length, cm	3.72	4.06	3.79	3.71	0.09	0.137	0.149	0.029
Proventricular length, cm/g OW	0.79	0.76	0.85	0.71	0.04	0.899	0.056	0.247
Proventricular width, cm	1.66	1.73	1.64	1.73	0.05	0.839	0.134	0.855
Proventricular width, cm/g OW	0.35	0.33	0.36	0.33	0.01	0.373	0.068	0.758
Gizzard length, cm	4.69	4.78	4.73	4.83	0.16	0.768	0.544	0.992
Gizzard length, cm/g OW	0.32	0.33	0.34	0.32	0.02	0.945	0.987	0.437
Gizzard width, cm	2.98	3.14	3.13	3.14	0.08	0.381	0.341	0.395
Gizzard width, cm/g OW	0.21	0.22	0.22	0.21	0.01	0.797	0.951	0.317
Main gizzard muscles^5^, cm	2.25	2.21	2.21	2.05	0.12	0.437	0.426	0.635
Main gizzard muscles, cm/g OW	0.16	0.16	0.16	0.14	0.01	0.493	0.371	0.485

1 *Expanded and pelleted*.

2 *Finely ground and pelleted*.

3 *Technical feed treatment*.

4 *Organ weight*.

5 *Main gizzard muscles = thickness of the musculus crassus cranioventralis (cm) + thickness of musculus crassus caudodorsalis (cm)*.

a−c *Different superscripts within a row mark significant differences between feeding groups (P <0.05)*.

In general, the pooled LsMeans of heart weights were significantly higher in broilers fed expanded and pelleted feed compared to broilers fed finely ground and pelleted feed (*P* = 0.002). Moreover, heart weights were significantly increased in broilers fed expanded and pelleted feed containing 6% RSE compared to heart weights of broilers fed finely ground and pelleted feed containing 12% RSE (*P* = 0.017). There was a significant negative correlation between the daily feed intake and the relative heart weight (*P* = 0.024) (Table 5).

**Table 5:** Significant correlations with performance parameters.

**Performance parameters**	**Correlated variables**	**Correlations coefficient (*r*)**	***P*-value**
ADFI^1^	Heart weight, g/kg BW^3^	−0.377	0.024
	Gizzard weight, g/kg BW	−0.357	0.032
	Gizzard length, cm	0.454	0.005
	Gizzard length, cm/g OW^4^	−0.389	0.019
	Gizzard width, cm/g OW	−0.488	0.003
	Thickness of main muscles, cm/g OW	−0.421	0.012
	Proventricular length, cm	0.515	0.001
	Proventricular length, cm/g OW	−0.597	<0.001
	Proventricular width, cm	0.493	0.002
	Proventricular width, cm/g OW	−0.665	<0.001
	pCO_2_^5^	−0.539	0.001
	TCO_2_^6^	−0.497	0.002
	${\text{HCO}}_{3}^{-7}$	−0.444	0.007
	Uric acid	−0.472	0.004
	CD3^+^/CD4^+^/CD8^+^ subsets of CT^8^	0.393	0.018
BWG^2^	Gizzard weight, g/kg BW	−0.412	0.012
	Gizzard length, cm	0.386	0.020
	Gizzard length, cm/g OW	−0.380	0.022
	Gizzard width, cm/g OW	−0.451	0.006
	Thickness of main muscles, cm/g OW	−0.380	0.022
	Proventricular length, cm	0.500	0.002
	Proventricular length, cm/g OW	−0.576	<0.001
	Proventricular width, cm	0.506	0.002
	Proventricular width, cm/g OW	−0.614	<0.001
	pCO_2_	−0.504	0.002
	TCO_2_	−0.472	0.004
	${\text{HCO}}_{3}^{-}$	−0.422	0.010
	Uric acid	−0.409	0.013
	CD3^+^/CD4^+^/CD8^+^ subsets of CT	0.349	0.037

1 *Average daily feed intake*.

2 *Body weight gain*.

3 *Body weight*.

4 *Organ weight*.

5 *Carbon dioxide partial pressure*.

6 *Total carbon dioxide*.

7 *Bicarbonate*.

8 *Caecal tonsils*.

Livers of broilers fed expanded and pelleted feed containing 12% RSE were significantly heavier compared to livers of broilers belonging to the feeding groups 6%ExP and 12%FgP (*P* < 0.05) and tended to be heavier compared to the livers of animals from the feeding group 6%FgP (*P* = 0.063).

The weight of bursa of *Fabricius* was significantly reduced after feeding expanded and pelleted feed containing 12% RSE compared to the weights of bursa of *Fabricius* of birds fed expanded and pelleted feed containing smaller amounts of RSE (*P* = 0.032).

The heaviest gizzards were found in birds fed expanded and pelleted feed containing 6% RSE, which were significantly heavier compared to gizzards of birds of the feeding groups 6%FgP and 12%ExP (*P* < 0.05). The lowest gizzard weights were measured in animals fed finely ground and pelleted feed containing 6% RSE. These values differed significantly from gizzard weights of birds fed other feeds (*P* < 0.05) except for gizzard weights of birds belonging to the feeding group 12%ExP. Additionally, significantly higher proventricular weights were measured in broilers of the feeding groups 6%ExP and 12%FgP compared to broilers of the two other feeding groups (*P* < 0.05). Considering the pooled LsMeans of the gizzard/proventriculus ratio, a higher ratio was shown in birds fed expanded and pelleted feed compared to birds fed finely ground and pelleted feed (*P* = 0.018). Furthermore, the pooled LsMeans of proventricular length and width related to g organ weight tended to be higher in animals fed expanded and pelleted feed compared to their counterparts (*P* < 0.1).

There were significant correlations between the daily feed intake and the gizzard weight as well as the length and the width of this organ (only width in cm per g organ weight) (*P* < 0.05). Moreover, the daily feed intake correlated significantly with the thickness of main gizzard muscles (*P* = 0.011). Additionally, there were significant correlations between the daily feed intake and the proventricular length and width (*P* < 0.05).

### Hematological Traits

The pooled LsMeans of the hematocrit of birds fed expanded and pelleted feed were significantly higher compared the pooled LsMeans of birds fed finely ground and pelleted feed (*P* = 0.007) (Table 6).

**Table 6:** Hematological traits assessed for broilers in the different feeding groups (LsMeans; *n* = 9).

**Variable**	**6% rapeseed expeller**		**12% rapeseed expeller**		**SE**	***P*****-value**		
	**ExP^1^**	**FgP^2^**	**ExP**	**FgP**		**Diet**	**TFT^3^**	**Diet x TFT**
Hematocrit, %	30.8	25.9	30.3	27.5	1.3	0.704	0.007	0.445
Leucocytes, cells/μL	23,891	26,115	20,733	19,171	3,829	0.196	0.932	0.624
Thrombocytes, cells/μL	13,878	6,264	8,393	7,104	2,642	0.386	0.102	0.240
Lymphocytes, %	47.7	54.5	54.4	53.4	3.6	0.448	0.426	0.287
Heterophilic granulocytes, %	45.2	39.8	39.8	40.6	3.1	0.473	0.468	0.324
H/L ratio^4^	1.21	1.49	1.51	1.48	0.2	0.507	0.588	0.494
Eosinophilic granulocytes, %	3.72	2.44	2.83	2.22	0.57	0.339	0.108	0.564
Basophilic granulocytes, %	3.17	2.78	2.86	2.61	0.48	0.625	0.509	0.885
Monocytes, %	0.64^ab^	0.42^b^	0.25^b^	0.97^a^	0.13	0.515	0.057	0.001

1 *Expanded and pelleted*.

2 *Finely ground and pelleted*.

3 *Technical feed treatment*.

4 *Heterophilic granulocyte/lymphocyte ratio*.

a−b *Different superscripts within a row mark significant differences between feeding groups (P <0.05)*.

The proportions of monocytes were significantly higher in animals fed finely ground and pelleted feed containing 12% RSE compared to proportions in animals of the feeding groups 6%FgP and 12%ExP (*P* < 0.05). Proportions of other leucocytes were not influenced by the fixed factors (*P* > 0.05).

### Blood Gases and Electrolytes

The pooled LsMeans of the pO_2_ tended to be lower in broilers fed 12% RSE compared to broilers fed a diet containing lower amounts RSE (*P* = 0.055) (Table 7). Furthermore, the pooled LsMeans of the pO_2_ tended to be lower in birds fed expanded and pelleted feed compared to birds fed finely ground and pelleted feed (*P* = 0.067). The pooled LsMeans of TCO_2_ and ${\text{HCO}}_{3}^{-}$ levels tended to be higher in birds fed feeds containing 12% RSE than TCO_2_ and ${\text{HCO}}_{3}^{-}$ levels of birds fed lower amounts of RSE (*P* < 0.1).

**Table 7:** Blood gas traits and electrolytes assessed for broilers in the different feeding groups (LsMeans, *n* = 9).

**Variable**	**6% rapeseed expeller**		**12% rapeseed expeller**		**SE**	***P*****-value**		
	**ExP^1^**	**FgP^2^**	**ExP**	**FgP**		**Diet**	**TFT^3^**	**Diet x TFT**
Blood gas traits^4^								
pH	7.20	7.22	7.23	7.21	0.02	0.722	0.968	0.408
pCO_2_, mmHg^5^	68.9	63.9	68.3	68.6	3.8	0.596	0.538	0.491
pO_2_, mmHg^6^	54.6	58.4	40.6	54.1	4.6	0.055	0.067	0.302
TCO_2_, mmol/L^7^	26.9	26.4	28.1	27.6	0.6	0.071	0.407	0.997
BE_ecf_, mmol/L^8^	−1.86	−1.99	−0.26	−1.10	0.75	0.109	0.523	0.643
${\text{HCO}}_{3}^{-}$, mmol/L^9^	25.2	24.8	26.4	25.8	0.6	0.066	0.422	0.923
Electrolytes, mmol/L								
Na^+^	147.8	146.9	146.7	148.1	0.5	0.960	0.650	0.036
K^+^	4.86	4.58	4.39	4.77	0.24	0.562	0.834	0.176
Na^+^/K^+^ ratio	31.4	32.4	34.6	31.5	1.65	0.485	0.518	0.227
Cl^−^	109.8	109.3	108.8	109.4	0.8	0.568	0.919	0.481
Ca^2+^	1.49^b^	1.44^b^	1.61^a^	1.48^b^	0.03	0.011	0.004	0.159
Anion gap	17.7	17.4	15.9	17.6	0.90	0.398	0.443	0.278

1 *Expanded and pelleted*.

2 *Finely ground and pelleted*.

3 *Technical feed treatment*.

4 *Blood gases represented as body temperature-corrected values*.

5 *Carbon dioxide partial pressure*.

6 *Oxygen partial pressure*.

7 *Total carbon dioxide*.

8 *Base concentration in extracellular fluid*.

9 *Bicarbonate*.

a, b *Different superscripts within a row mark significant differences between feeding groups (P <0.05)*.

The blood gas parameters TCO_2_, ${\text{HCO}}_{3}^{-}$, and pCO_2_ were negatively correlated with daily feed intake and the BW gain (*P* < 0.05) (Table 5). Furthermore, the length and the width (both in cm per g organ weight) of proventriculus correlated significantly with the blood gas parameters, pCO_2_, TCO_2_, and ${\text{HCO}}_{3}^{-}$ (*r* > 0.40; *P* < 0.05).

The calcium concentration in blood was significantly affected by both fixed effects (*P* < 0.05). Concretely, the highest calcium concentrations were detected in broilers fed expanded and pelleted feed containing 12% RSE, which were significantly higher compared to calcium concentrations of the broilers from other feeding groups (*P* < 0.05).

### Blood Metabolites and Liver Enzymes

The pooled LsMeans of cholesterol concentrations of broilers fed 12% RSE were significantly higher compared to the LsMeans of broilers fed lower amounts of RSE (*P* = 0.050) (Table 8). Furthermore, the pooled LsMeans of albumin concentrations and aspartate-amino-transferase activity tended to be higher in broilers fed 12% RSE compared to LsMeans of animals fed lower amounts of RSE (*P* < 0.1).

**Table 8:** Blood metabolite concentrations and liver enzyme activities assessed for broilers in the different feeding groups (LsMeans, *n* = 9).

**Variable**	**6% rapeseed expeller**		**12% rapeseed expeller**		**SE**	***P*****-value**		
	**ExP^1^**	**FgP^2^**	**ExP**	**FgP**		**Diet**	**TFT^3^**	**Diet x TFT**
Albumin, g/L	13.5	13.1	14.5	13.8	0.4	0.057	0.223	0.669
Total protein, g/L	22.2	19.3	22.5	22.3	1.0	0.105	0.128	0.169
Glucose, mg/dL	234.1	227.0	228.0	228.3	6.7	0.726	0.619	0.585
Lactate, mmol/L	7.63	7.10	6.80	7.84	0.66	0.950	0.698	0.242
Cholesterol, mg/dL	131.4	129.4	150.3	133.8	5.7	0.050	0.116	0.211
Aspertate-amino-transferase, IU/L	154.3	145.7	170.4	159.2	7.4	0.055	0.193	0.858
γ-Glutamyltransferase, IU, L	20.9	20.7	21.8	21.0	0.9	0.526	0.607	0.698
Triglycerides, mg/dL	76.0	65.7	78.1	80.5	6.7	0.217	0.561	0.354
Urea, mg/dL	6.69	4.92	5.95	11.76	2.70	0.266	0.459	0.170
Uric acid, mg/dL	5.68^a^	4.37^b^	5.17^ab^	5.59^ab^	0.33	0.296	0.188	0.014
β-Hydroxybutyrate, mmol/L	0.63	0.63	0.62	0.71	0.06	0.552	0.447	0.478
NEFA^4^, mmol/L	0.70	0.70	0.78	0.65	0.05	0.827	0.258	0.201

1 *Expanded and pelleted*.

2 *Finely ground and pelleted*.

3 *TFT = technical feed treatment*.

4 *Non-esterified fatty acids*.

a, b *Different superscripts mark significant differences between feeding groups (P <0.05)*.

The blood uric acid concentration in broilers fed expanded and pelleted feed containing 6% RSE was significantly higher compared to the concentration of broilers fed finely ground and pelleted feed containing similar amounts of RSE (*P* = 0.041). The uric acid concentration was significantly negatively correlated with the daily feed intake (*P* = 0.004) (Table 5) and significantly positively correlated with the hematocrit (*r* = 0.516; *P* = 0.001).

### Subsets of T and B Cells

The pooled LsMeans of proportions of CD3^+^/CD4^−^/CD8^−^ stained PBLs in birds fed expanded and pelleted feed were significantly lower compared to pooled LsMeans in birds fed finely ground and pelleted feed (*P* = 0.048) (Table 9). Moreover, the pooled LsMeans of proportions of CD3^+^/CD4^+^/CD8^+^ stained PBLs in birds fed expanded and pelleted feed tended to be lower compared to pooled LsMeans in birds fed finely ground and pelleted feed (*P* = 0.090). The pooled LsMeans of CD3^+^/CD4^+^/CD8^−^ subsets of PBLs tended to be lower in birds fed 12% RSE compared to the pooled LsMeans of birds fed lower amounts of RSE (*P* = 0.070).

**Table 9:** T cell subsets in blood, *lamina propria*, and caecal tonsils (LsMeans ± SE, *n* =9).

**Variable**	**6% rapeseed expeller**		**12% rapeseed expeller**		***P*****-value**		
	**ExP^1^**	**FgP^2^**	**ExP^4^**	**FgP**	**Diet**	**TFT^3^**	**Diet x TFT**
Blood
CD3^+^/CD4^+^/CD8^−^, %	57.6 ± 2.2	52.8 ± 2.2	50.9 ± 2.3	51.3 ± 2.2	0.070	0.336	0.249
CD3^+^/CD4^−^/CD8^+^, %	26.7 ± 2.2	29.3 ± 2.2	33.2 ± 2.4	27.8 ± 2.2	0.282	0.552	0.085
CD3^+^/CD4^+^/CD8^+^, %	1.7 ± 0.5	3.3 ± 0.5	2.8 ± 0.6	3.1 ± 0.5	0.443	0.090	0.286
CD3^+^/CD4^−^/CD8^−^, %	14.0 ± 1.2	14.5 ± 1.2	13.3 ± 1.3	17.8 ± 1.2	0.311	0.048	0.115
*Lamina propria*
CD3^+^/CD4^+^/CD8^−^, %	21.2 ± 2.9	16.0 ± 2.9	15.2 ± 2.9	13.8 ± 2.9	0.166	0.263	0.508
CD3^+^/CD4^−^/CD8^+^, %	34.5 ± 4.4	40.6 ± 4.4	36.5 ± 4.4	42.4 ± 4.4	0.671	0.185	0.983
CD3^+^/CD4^+^/CD8^+^, %	9.0 ± 3.3	9.1 ± 3.3	18.4 ± 3.3	15.3 ± 3.3	0.024	0.657	0.633
CD3^+^/CD4^−^/CD8^−^, %	35.3 ± 3.0	34.3 ± 3.0	29.9 ± 3.0	28.5 ± 3.0	0.074	0.688	0.943
Caecal tonsils
CD3^+^/CD4^+^/CD8^−^, %	25.2 ± 3.5	25.3 ± 3.5	20.0 ± 3.5	22.1 ± 3.5	0.240	0.751	0.777
CD3^+^/CD4^−^/CD8^+^, %	59.3 ± 4.4	60.0 ± 4.4	61.8 ± 4.4	60.4 ± 4.4	0.744	0.940	0.812
CD3^+^/CD4^+^/CD8^+^, %	2.3 ± 0.3	2.9 ± 0.3	2.9 ± 0.3	2.6 ± 0.3	0.625	0.748	0.199
CD3^+^/CD4^−^/CD8^−^, %	13.2 ± 2.4	11.8 ± 2.4	15.3 ± 2.4	14.9 ± 2.4	0.300	0.714	0.839

1 *Expanded and pelleted*.

2 *Finely ground and pelleted*.

3 *Technical feed treatment*.

4 *Blood n = 8*.

The pooled LsMeans of CD3^+^/CD4^+^/CD8^+^ subsets in the *lamina propria* were significantly higher in broilers fed feeds containing 12% RSE compared to the pooled LsMeans of broilers fed feeds containing lower amounts of RSE (*P* = 0.024). Furthermore, considering the pooled LsMeans the CD3^+^/CD4^−^/CD8^−^ subsets tended to be less frequent in the *lamina propria* of broilers fed 12% RSE compared broilers fed 6% RSE (*P* = 0.074).

There were no significant effects of diet or TFT on Bu^+^ subsets in blood and *lamina propria* (*P* > 0.05) (data not shown). No significant influences of fixed effects on the T cell subsets were detected in caecal tonsils (*P* > 0.05).

There was a significant correlation between the proventricular weight and the CD3^+^/CD4^+^/CD8^+^ subsets in caecal tonsils (*r* = −0.404; *P* = 0.014) as well as between the gizzard length (cm) and the CD3^+^/CD4^+^/CD8^−^ T cell subsets in *lamina propria* (*r* = −0.374; *P* = 0.025). Additionally, CD3^+^/CD4^+^/CD8^+^ subsets correlated significantly with the daily feed intake (*P* = 0.017) (Table 5).

### Principal Component Analysis

**Figure 1:**
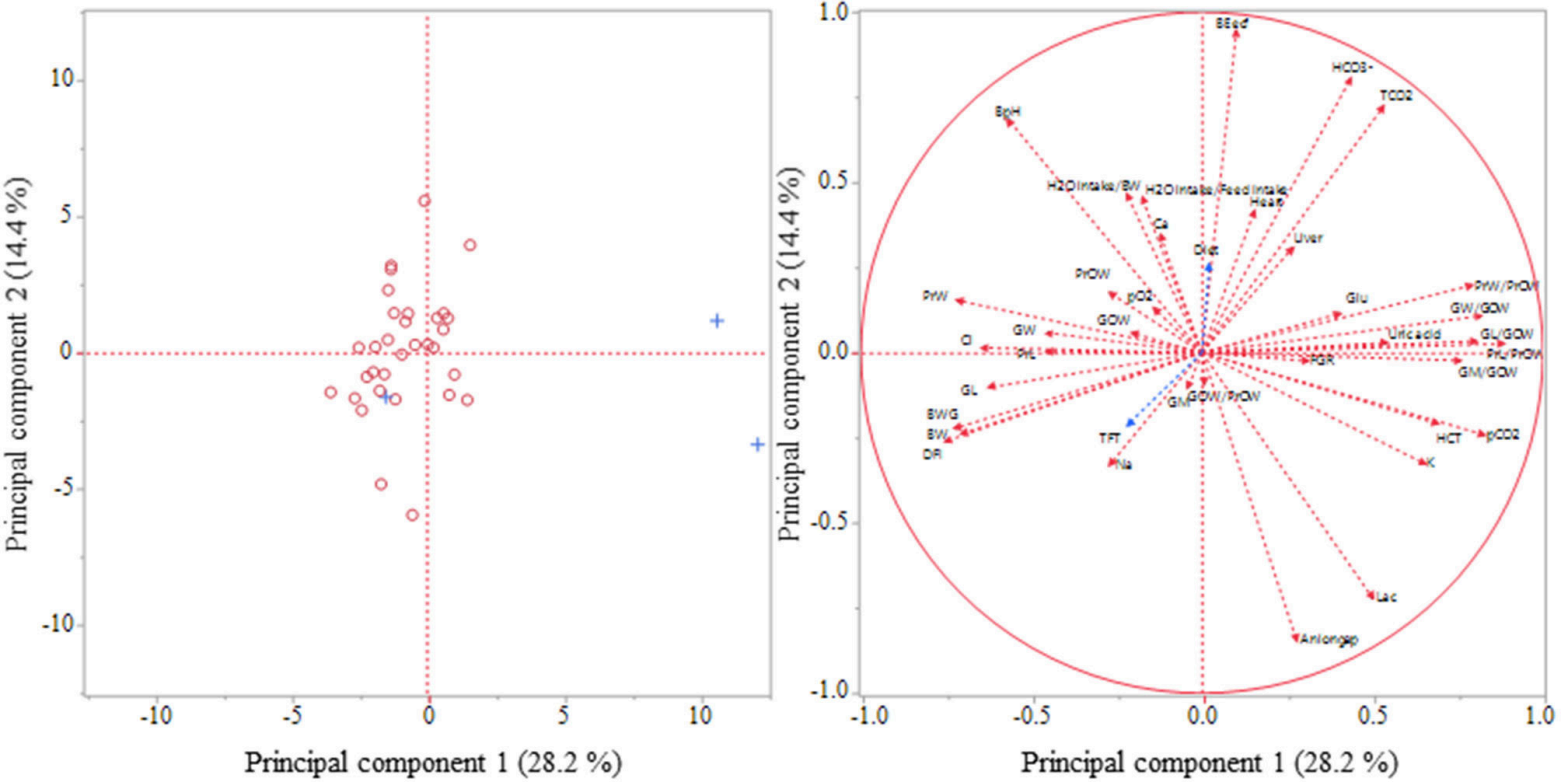
Principal Component Analysis for visualization of the relationships between 38 selected variables. Variables for the analysis: DFI, daily feed intake; BW, body weight on slaughtering day; BWG, body weight gain; FGR, feed to gain ratio; H2O intake/BW, H_2_O consumption per kg body weight; H2O intake/Feed intake, H_2_O consumption per g feed intake; Heart, relative heart weight; Liver, relative liver weight; PrOW, relative organ weight of proventriculus; PrL, proventricular length (cm), PrL/PrOW, proventricular length (cm/g organ weight); PrW, proventricular width (cm); PrW/PrOW, proventricular width (cm/g organ weight); GOW, relative gizzard weight; GOW/PrOW, gizzard weight/proventricular weight ratio; GL, gizzard length (cm), GL/GOW, gizzard length (cm/g organ weight); GW, gizzard width (cm); GW/GOW, gizzard width (cm/g organ weight); GM, gizzard muscles (cm); HCT, hematocrit; BpH, blood pH; pO2, oxygen partial pressure in blood; pCO2, carbon dioxide partial pressure in blood; TCO2, total carbon dioxide concentration in blood; BEecf, base concentration in extracellular fluid; HCO3-, bicarbonate concentration in blood; Na, sodium concentration in blood; K, potassium concentration in blood; Cl, chloride concentration in blood; Ca, ionized calcium concentration in blood; Aniongap, anion gap; Glu, glucose concentration in blood; Lac, lactate concentration in blood; Uric acid, uric acid concentration in blood. Additional variables: Diet; TFT, technical feed treatment; Left: projection of cases (broilers): + with diagnosed ascites; O without diagnosed ascites; Right: projection of variables.

A principal component analysis was performed to visualize possible relationships between 38 variables belonging to broiler performance, organ traits, blood gases, blood electrolytes, and blood metabolites (Figure 1). The analysis revealed the first two principal components, which extracted ~ 42.6% of the total variance. According to a scree plot and the eigenvalue consecutive principal components were assessed. The mean eigenvalue of 1.0 of all components corresponded to a total of nine extracted components, which explained cumulatively 86.6% of the total variance between the 38 variables.

Several variables appeared to correlate strongly to the principal component 1 and 2 indicated by their close localization to the outer circle of the graph (Figure 1). Only variables such as FGR, gizzard weight, gizzard weight/proventricular weight ratio, gizzard main muscle thickness, and the pO_2_ value showed a closer localization to the center of the cross indicating low correlations to the considered principal component. Also the additional variables diet and TFT were closely located to the center of the cross rather than to the outer circle.

The performance parameters, daily feed intake, BW gain, and BW on slaughtering day clustered with each other and appeared to correlate highly negatively with both principal components. In contrast, blood gas parameters TCO_2_ and ${\text{HCO}}_{3}^{-}$ and the organ traits, proventricular and gizzard width (cm/g organ weight), as well as proventricular and gizzard length (cm/g organ weight) showed an opposite direction to the mentioned performance parameters.

## Discussion

The present study investigated the interactive effects between dietary rapeseed proportion and technical treatments of diets, which included expanding and pelleting or fine grinding prior to pelleting on broiler performance, morphometric traits of the proventriculus, the gizzard and other selected visceral organs, as well as immunological traits in broilers.

Broilers fed expanded and pelleted feed showed reduced daily feed intake compared to broilers fed finely ground and pelleted feed when feeding a 6% RSE diet. However, there was no difference between the daily feed intake of birds fed differently processed feeds containing higher amounts of RSE. Perhaps, these differences were a result of the different pellet qualities, which were indicated by the pellet abrasion. It appears that a higher pellet abrasion resulted in a lower daily feed intake. A relationship between pellet quality and broiler performance was also reported in studies of Lilly et al. (20). Furthermore, the differences in pellet abrasion might be also an indication for modified pellet textures due to the various feed ingredients and processing methods. The importance of pellet texture in broiler performance was also shown in studies of Parsons et al. (11). The reason for the differences in pellet abrasion of our current feeds appeared to be related to the particle size distribution before hydrothermal treatment. The influences of feed on daily feed intake were also reflected in the BW gain, which in turn influenced the BW on slaughtering day.

The water consumption was markedly influenced by diet composition. Also in studies of Abd El-Wahab et al. (21) differences in water consumption were observed between broilers fed a soybean meal based diet and broilers fed a diet based on rapeseed meal. An effect of total protein content on water consumption as reported by Wheeler and James (22) can be excluded because the total protein content did not differ between the feeds, which was published by Liermann et al. (23). Thus, in general the water consumption seemed to depend on protein sources in the diet. The significant diet-by-TFT interaction detected in case of the water to feed ratio demonstrated that the TFT has also an impact on the water consumption. This suggestion is supported by the results of Vranjes and Wenk (24) showing an increased water consumption after feeding extruded feed components compared to only pelleted feed components. Manning et al. (25) emphasized that the water consumption is directly related with the fecal conditions. In turn, this could affect the litter quality and the incidence of foot pad dermatitis (25). In studies of Abd El-Wahab et al. (21) a higher incidence of foot pad dermatitis after feeding rapeseed meal seemed to be also associated with a higher water consumption of broilers compared to broilers fed soybean meal. However, in contrast to present results in these studies the water/feed ratio of birds fed rapeseed meal was numerically decreased compared to birds fed soybean meal.

Significantly higher liver weights and numerically enhanced cholesterol and non-esterified fatty acids concentrations as well as aspartate-amino-transferase activities indicated alterations in the liver metabolism of birds fed expanded and pelleted feed containing 12% RSE compared to birds fed the other tested feeds. The observed alterations in liver weights and blood cholesterol concentrations as well as the higher ionized calcium concentrations in these birds indicated also changes in the calcium metabolism. Cholesterol is a precursor of vitamin D_3_ and both are metabolized in the liver (26, 27). In turn, vitamin D_3_ and its metabolites play a key role in the absorption of calcium in the intestine and the kidneys and the incorporation in bones (28). The reasons for the apparent alterations in liver and calcium metabolism cannot be fully explained currently. In part these results appear to be diet-dependent. However, broilers fed finely ground and pelleted feed containing 12% RSE content showed no comparable alterations. Besides the diet also the TFT affected ionized calcium concentrations in blood. Moreover, there was a significant diet-by-TFT interaction in case of liver weights. Thus, also the TFT appears to play a role in the development of the effects on liver and calcium metabolism. Differences in blood calcium concentrations might be also possible by differences in vitamin D_3_ supplementation. It is known, that vitamin D_3_ stability varies between pelleting and expanding (29). A possible destruction of the vitamin in the premix during feed processing might result in different vitamin D_3_ content in feed and therefore in differences in vitamin intake. The aspect that feeds processed by similar methods did not show similar effects on blood calcium concentrations may contradict this hypothesis. However, it has to be emphasized, that the extent of the impacts of TFT on the feed characteristics depends strongly on feed components, ingredients, and structure of feed material (30, 31). Because albumin acts as a calcium carrier it is not surprising that this serum protein was affected in a similar manner as calcium.

Different previous studies revealed that feeding compacted feed plays a key role in the development of proventricular dilatation and underdevelopment of the gizzard (4, 5, 32). Comparing gizzard weights and the proventricular size (length and width) measured in the present study with similar organ traits measured in broilers fed coarsely ground meal in a previous study of Liermann et al. (5), it is suggested that the adverse effects of pelleted feed on the development of the proventriculus or the gizzard were not markedly inhibited by any of the used pre-treatments or diets, although the gizzard and the proventricular weights differed between the feeding groups and were affected in a diet-dependent manner. The study of Liermann et al. (5) and the current study demonstrated that the proventricular extent appears to be a more reliable indicator for proventricular dilatation than the weight of this organ, because the increase in organ size is not associated with an increase in organ muscle or tissue growth but by stretching of this organ. Significant correlations between the daily feed intake and some morphometric traits of the digestive organs indicated that differences in the development of the proventriculus and the gizzard are strongly related with the daily feed intake. This aspect was also supported by findings of Liermann et al. (5) and in turn explains the differences in gizzard and proventricular traits between the current feeding groups. As discussed by Svihus (33) one main function of the gizzard is the feed intake regulation. Because of the underdevelopment of the gizzard by feeding pellets and by covering the satiety signal by the appetite in modern broiler lines (33), an overconsumption will be supported, which leads to proventricular dilatation.

In the present study the risk for pulmonary hypertension and ascites seemed to be neither increased nor markedly decreased in the different feeding groups, although the feeding groups differed markedly in animal performance. In the literature, fast growing by a high nutrient intake and overconsumption of feed was associated with a higher incidence of ascites (7, 34, 35). So, Baghbanzadeh and Decuypere (7) suggested feed restriction as a successful method to reduce ascites. Contrary to the expectations, birds of the 6%FgP group showing the highest daily feed intake and BW gain were not associated with a higher occurrence of this metabolic disease at an age of 28 days after hatching. Additionally, one animal showing low fattening performance and belonging to feeding group 6%ExP suffered from ascites. Indeed, ascites is based on multifactorial causes as also reported by Baghbanzadeh and Decuypere (7). Ascites is characterized by hypoxemia, hypercapnia, right ventricular hypertrophy, and an elevated hematocrit (7, 35). Therefore, it was assumed that fast growing broilers might show similar alterations. However, pCO_2_ and TCO_2_ levels, heart weights, as well as the hematocrit were negatively correlated to the daily feed intake and in part to the BW gain. Previous studies also reported relationships between performance parameters and, for example, hematocrit values or blood gas values, although these parameters were in part affected in an opposite direction (35, 36). Ascites was only diagnosed in three animals during slaughtering. However, the principal component analysis shows that birds suffering from ascites markedly differed in variables considered in the principal component analysis except for one animal. In contrast, all other animals clustered with each other. It cannot be excluded that some further broilers would develop ascites at a later time. Using the hematocrit as a selection criteria as reported by Baghbanzadeh and Decuypere (7), birds fed expanded and pelleted feed might have had a higher risk for ascites, and this risk is more related on the TFT than on the diet.

Ascites is associated with oxidative stress in birds (7). Uric acid is a potent scavenger of reactive oxygen species, and it was shown in studies of Simoyi et al. (37) that a decrease in uric acid concentrations in plasma is associated with a marked increase of oxidative stress. In contrast in studies of Enkvetchakul et al. (38) ascites increased uric acid concentrations in blood, lung, and liver. In the present study broilers suffering from ascites showed higher uric acid concentrations compared to broilers without ascites (data not shown). Furthermore, the uric acid concentration was positively correlated with the hematocrit. According to these findings possibly a higher uric acid concentration might be also a distinguishing feature for birds prone to ascites. Using both the hematocrit and the uric acid concentration as distinguishing feature, especially animals from the 6%ExP group might have a higher risk for ascites in the present study. This hypothesis should be tested in further studies with a higher number of animals.

Already, in studies of Liermann et al. (5) it was suggested that the alterations in proventriculus and gizzard could also increase the incidence of pulmonary hypertension and ascites. Both the correlations between the traits of the digestive organs and the blood gases as well as the principal component analysis clearly showed that there are some relationships between these factors indicating a possible impact of the development of the digestive tract and functions of pulmonary gas exchange.

Interestingly, PBL proportions of CD4/CD8 double positive and double negative T cells were significantly affected or tended to be affected by the TFT. In contrast, these cell types were significantly influenced or tended to be influenced by the diet type in *lamina propria*. It was assumed that the effects on the peripheral system are a result of the nutrient absorption and possible changes in blood metabolites. However, the mentioned subsets correlated neither with the daily feed intake nor with blood metabolites. However, there was a significant correlation between the digestibility of crude fiber [as presented in Liermann et al. (23)] and the PBL proportions of CD4/CD8 double negative stained T cells (*r* = −0.371; *P* = 0.028). The fiber digestibility was also affected by the TFT (23). During the digestion of fiber in the caeca by microbes different metabolites and especially short chain fatty acids are produced. These metabolites are often discussed as immune-modulators (39). The effects of the diet on T cell subsets in the *lamina propria* might be a local effect possibly based on changes on physico-chemical conditions in the intestinal content. The formulated diets showed no marked differences in crude nutrients and were free from glucosinolates as published by Liermann et al. (23). Therefore, these aspects can be excluded as a potential impact factor. This applies also to the trend to affect PBL CD3^+^/CD4^+^/CD8^−^ T cell subsets by diet. The biological importance of these findings needs to be proved in further studies. In general, both the CD4/CD8 double positive and the double negative T cells are associated with immune-regulatory activities (40, 41); however, the role of these cells has not been fully explored, especially in poultry. The found correlations between morphometric traits of gizzard and proventriculus and T cell subsets in *lamina propria* and caecal tonsils in studies of Liermann et al. (5) and in the current study let assume that alterations in the development of organs of the proximal digestive tract are associated with modifications in the local immune system of the distal digestive tract. Similar observations have been made in studies with pigs of Liermann et al. (42). Interestingly and similar to studies of Liermann et al. (5), the T cell subsets that show relationships to gizzard or proventricular weight show also correlations to the daily feed intake.

The differences in the relative weights of bursa of *Fabricius* between the feeding groups 6%ExP and 12%ExP cannot be explained at the moment. In previous studies decreasing weights of this organ was associated with an immunosuppression and as an indicator for stress. However, as also reported in studies of Liermann et al. (5) this parameter did not correspond to the H/L ratio, which was not different between the feeding groups and is also accepted as a stress indicator in chickens (43).

Because of the low frequency of monocytes in blood in the current study, the biological relevance of the observed differences between feeding groups seemed to be unlikely.

## Conclusion

In conclusion, the pellet quality and texture that are mainly influenced by the feed processing methods seemed to play a key role in the development of the gizzard and the proventriculus because of their influences on the daily feed intake. Alterations in morphometric traits of proventriculus and gizzard modify the local immune system of the downstream digestive tract and might have influences on the development of ascites. However, further studies are required to confirm this hypothesis since in the present study only three birds developed ascites.

## Data Availability Statement

The raw data supporting the conclusions of this article will be made available by the authors, without undue reservation.

## Ethics Statement

The animal study was reviewed and approved by Lower Saxony State Office for Consumer Protection and Food Safety, LAVES, Germany (registration number: 33.9-42502-04-082/09).

## Author Contributions

WL, AB, VB, and SD: conceptualization. WL and JF: methodology. WL, JF, and AB: investigation. VB, AB, and SD: resources. WL: writing-original draft preparation. AB, JF, and SD: writing-review and editing. SD: supervision. AB and VB: project administration and funding acquisition. All authors contributed to the article and approved the submitted version.

### Conflict of Interest

The authors declare that the research was conducted in the absence of any commercial or financial relationships that could be construed as a potential conflict of interest.

## Abbreviations

ExP: expanded and pelleted
FgP: finely ground and pelleted
RSE: rapeseed expeller
TFT: technical feed treatment
PBL: peripheral blood leucocytes
RMPI-1640: Roswell Park Memorial Institute medium
BW: body weight
FGR: feed to gain ratio
H/L ratio: heterophilic granulocytes to lymphocyte ratio
LSMeans: least squares means.
